# The PRR11-SKA2 Bidirectional Transcription Unit Is Negatively Regulated by p53 through NF-Y in Lung Cancer Cells

**DOI:** 10.3390/ijms18030534

**Published:** 2017-03-01

**Authors:** Yitao Wang, Huali Weng, Ying Zhang, Yinjiang Long, Yi Li, Yulong Niu, Fangzhou Song, Youquan Bu

**Affiliations:** 1 Department of Biochemistry and Molecular Biology, Chongqing Medical University, 1# Yixueyuan Road, Yuzhong District, Chongqing 400016, China; wytao8899@126.com (Y.W.); wyinying@aliyun.com (H.W.); vera21173@163.com (Y.Z.); maggie8818@sina.com (Y.L.); cecilylee@126.com (Y.L.); fzsongcq@163.com (F.S.); 2 Molecular Medicine and Cancer Research Center, Chongqing Medical University, Chongqing 400016, China; yulong.niu@aol.com

**Keywords:** *p53*, NF-Y, bidirectional promoter, *PRR11*, *SKA2*, lung cancer

## Abstract

We previously identified *proline-rich protein 11* (*PRR11*) as a novel cancer-related gene that is implicated in the regulation of cell cycle and tumorigenesis. Our recent study demonstrated that *PRR11* and its adjacent gene, *kinetochore associated 2* (*SKA2*), constitute a classic head-to-head gene pair that is coordinately regulated by nuclear factor Y (NF-Y). In the present study, we further show that the *PRR11-SKA2* bidirectional transcription unit is an indirect target of the tumor suppressor p53. A luciferase reporter assay revealed that overexpression of wild type *p53*, but not mutant *p53*, significantly represses the basal activity and NF-Y mediated transactivation of the *PRR11-SKA2* bidirectional promoter. Deletion and mutation analysis of the *PRR11-SKA2* promoter revealed that p53-mediated *PRR11-SKA2* repression is dependent on the presence of functional NF-Y binding sites. Furthermore, a co-immunoprecipitation assay revealed that p53 associates with NF-Y in lung cancer cells, and a chromatin immunoprecipitation assay showed that p53 represses *PRR11-SKA2* transcription by reducing the binding amount of NF-Y in the *PRR11-SKA2* promoter region. Consistently, the ability of p53 to downregulate *PRR11-SKA2* transcription was significantly attenuated upon siRNA-mediated depletion of nuclear factor Y subunit beta (*NF-YB*). Notably, lung cancer patients with lower expression of either *PRR11* or *SKA2* along with wild type *p53* exhibited the best overall survival compared with others with *p53* mutation and/or higher expression of either *PRR11* or *SKA2*. Taken together, our results demonstrate that p53 negatively regulates the expression of the *PRR11-SKA2* bidirectional transcription unit through NF-Y, suggesting that the inability to repress the *PRR11-SKA2* bidirectional transcription unit after loss of p53 might contribute to tumorigenesis.

## 1. Introduction

Our previous study was the first to isolate and identify *proline-rich protein 11* (*PRR11*) as a novel cancer-related gene that is implicated in both cell cycle progression and lung cancer development [1,2]. Our data revealed that PRR11 is periodically expressed in a cell cycle-dependent manner. During the cell cycle, the expression level of PRR11 begins to increase at the late S phase, and remains high until telophase at which it quickly declines. Consistently, RNAi-mediated knockdown of *PRR11* causes significant S phase arrest and G2/M progression delay accompanied with obvious mitotic defects such as multipolar spindles and multiple nuclei. On the other hand, PRR11 expression is aberrantly upregulated at both mRNA and protein levels in primary lung cancer tissues compared with normal lung tissues. Higher expression of *PRR11* is significantly associated with poor prognosis in lung cancer patients. Consistently, knockdown of *PRR11* in lung cancer cells causes inhibition of proliferation, motility and colony formation ability accompanied with the dysregulation of multiple critical pathways and genes involved in the cell cycle, tumorigenesis and metastasis. Moreover, *PRR11* knockdown also reduces tumor growth in vivo in the xenograft nude mouse model of lung cancer [1,2]. Of note, two subsequent studies reported that PRR11 also has oncogenic potential and prognostic value in both gastric cancer and hilar cholangiocarcinoma, further demonstrating the critical role of PRR11 in both cell cycle progression and tumorigenesis [3,4].

Head-to-head gene pairs represent a distinct feature of gene organization, and account for more than 10% of genes in the human genome [5,6,7,8,9,10,11,12]. Two genes in a head-to-head gene pair usually have similar functions, and their expression is coordinately regulated. Transcription of such gene pairs is usually driven by a shared intergenic bidirectional promoter that initiates transcription in both orientations [5,6,7,8,9,10,13,14,15]. More recently, we further showed that *PRR11*, along with its upstream adjacent gene, *spindle and kinetochore associated 2* (*SKA2*), constitute a classic head-to-head gene pair driven by a prototypical bidirectional promoter containing CCAAT boxes [5]. Detailed analysis demonstrated that the *PRR11-SKA2* bidirectional transcription unit is a novel direct target of NF-Y that could directly binds to and transactivate the *PRR11-SKA2* bidirectional promoter in both orientations. Consistently, the elevated expressions of PRR11 and SKA2 in lung cancer are highly correlated with each other as well as with that of NF-Y. Univariate survival analyses revealed that lung cancer patients with high expression of both genes show poorer prognosis compared with that with only one or neither high expression. Functional analysis demonstrated that the *PRR11-SKA2* bidirectional transcription unit is essential for the accelerated proliferation and motility of lung cancer cells [5].

NF-Y, also known as CBF (CMP-binding factor) or CP1 (Cysteine proteinase-1), is a sequence-specific transcription factor that binds to the canonical CCAAT element and constitutes three subunits, NFYA, NFYB and NFYC [11]. To date, a large number of studies has shown that NF-Y plays a critical regulatory role in the expression of various genes implicated in proliferation, cell cycle progression, apoptosis and tumorigenesis [12,13,14,15,16,17]. Of note, p53 has been shown to regulate the expression of target genes by interacting with NF-Y and other transcription factors [15,17]. It is well known that *p53* is an important tumor suppressor gene, and more than 50% of human tumors are found to harbor mutations in the *p53* gene [18]. As a classic transcription factor, p53 plays a critical role in a variety of cellular processes including proliferation, cell cycle, senescence and apoptosis by regulating the expression of a large number of downstream target genes [19]. Given the established role of PRR11 and SKA2 in the cell cycle and tumorigenesis, the aim of this study is to investigate the potential regulatory effect of p53 on the *PRR11-SKA2* gene pair. Our present study found that p53 indirectly represses the *PRR11-SKA2* bidirectional transcription unit by interacting with the NF-Y transcription factor in lung cancer.

## 2. Results

### 2.1. p53 Represses PRR11-SKA2 Bidirectional Promoter Activity

**Figure 1:**
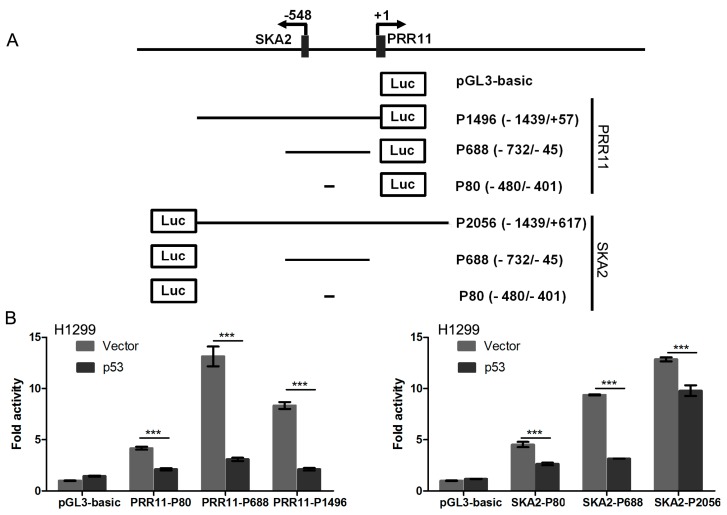
Overexpression of p53 represses *PRR11-SKA2* promoter activity. (**A**) Schematic representation of *PRR11-SKA2* bidirectional promoter reporter constructs. The positions relative to the major transcriptional initiation site of *PRR11* (+1) are indicated. The constructs were named as “P(promoter)-fragment length (start position/end position)”; (**B**) Luciferase reporter assay. H1299 cells were transiently co-transfected with the indicated luciferase reporter constructs together with *p53* expression vector by using Lipofectamine 2000 transfection reagent. Forty-eight hours after transfection, firefly and renilla luciferase activities were measured by Dual Luciferase Assay System (Promega, Madison, WI, USA). Data obtained from a representative of at least three independent experiments were shown as fold induction compared to the activity of cells transfected with the empty pGL3-basic vector. The results are presented as the mean and SD of triplicates from a representative experiment. *** *p* < 0.001.

To firstly determine whether p53 regulates the *PRR11-SKA2* bidirectional transcription unit, *p53*-deficient H1299 cells were co-transfected with the *p53* expression vector and a series of *PRR11-SKA2* luciferase reporters. The *PRR11-SKA2* bidirectional promoter fragment was inserted into pGL3 basic vectors in either *PRR11* or *SKA2* orientation. The luciferase reporter assay revealed that p53 overexpression caused a significant decrease in luciferase activity of all *PRR11*- and *SKA2*-orientated promoter reporter deletion constructs including the shortest constructs (PRR11-P80 and SKA2-P80) compared with the empty vector. Thus, these results clearly indicate that p53 represses the *PRR11-SKA2* bidirectional promoter in both *PRR11* and *SKA2* orientations (Figure 1).

### 2.2. p53 Represses the Endogenous Transcription of the PRR11-SKA2 Bidirectional Unit

**Figure 2:**
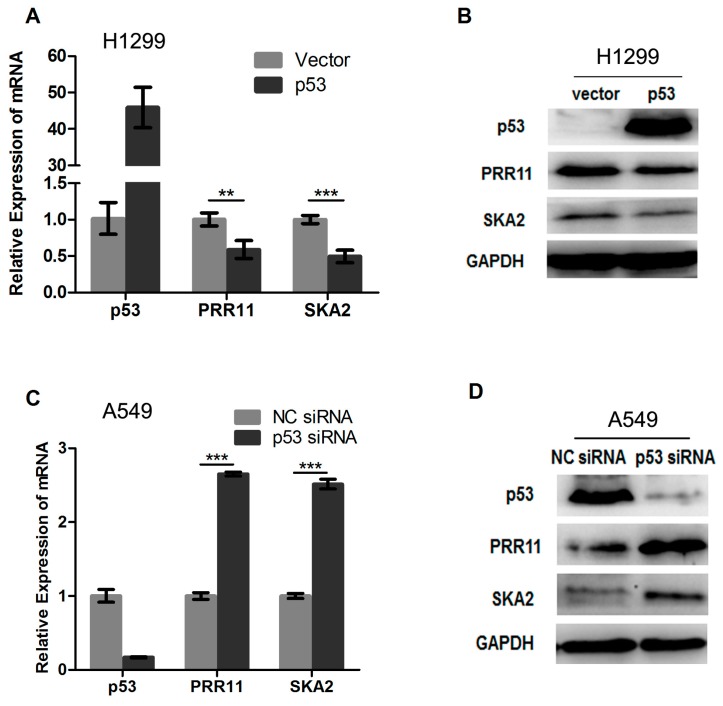
p53 represses endogenous *PRR11-SKA2* transcription. (**A**,**B**) Exogenous overexpression of *p53* represses endogenous PRR11 and SAK2 expression. H1299 cells (*p53*-deficient) were transiently transfected with the empty and *p53* expression vectors. Forty-eight hours after transfection, total RNA and whole cell lysates were prepared and subjected to qRT-PCR and Western blotting analyses. GAPDH was used as an internal control; (**C**,**D**) Knocking-down endogenous *p53* expression causes upregulation of *PRR11-SKA2*. A549 cells carrying wild type *p53* were transiently transfected with negative control (NC) or *p53* specific siRNAs. Forty-eight hours after transfection, cells were harvested, and total RNA and cell lysates were prepared and subjected to qRT-PCR and Western blotting analyses. ** *p* < 0.01, *** *p* < 0.001.

Next, we further determined whether p53 represses the endogenous transcription of *PRR11* and *SKA2* in cells. To this end, *p53*-deficient H1299 cells were transiently transfected with the wild type *p53* expression vector and control vector. As shown in Figure 2A,B, the exogenous expression of wild type *p53* resulted in significant repression of the endogenous PRR11 and SKA2 expression at both mRNA and protein levels. We then asked whether knockdown of the endogenous p53 could lead to the elevation of PRR11 and SKA2 expression. For this purpose, A549 cells carrying wild type *p53* were transiently transfected with specific siRNA targeting *p53* as well as negative control siRNA. As shown in Figure 2C,D, siRNA-mediated *p53* depletion resulted in an elevated endogenous expression of PRR11 and SKA2 at both mRNA and protein levels in cells. Taken together, these results suggest that p53 represses the endogenous transcription of the *PRR11-SKA2* bidirectional unit.

### 2.3. p53 Represses the Transcription of the PRR11-SKA2 Bidirectional Promoter through NF-Y

**Figure 3:**
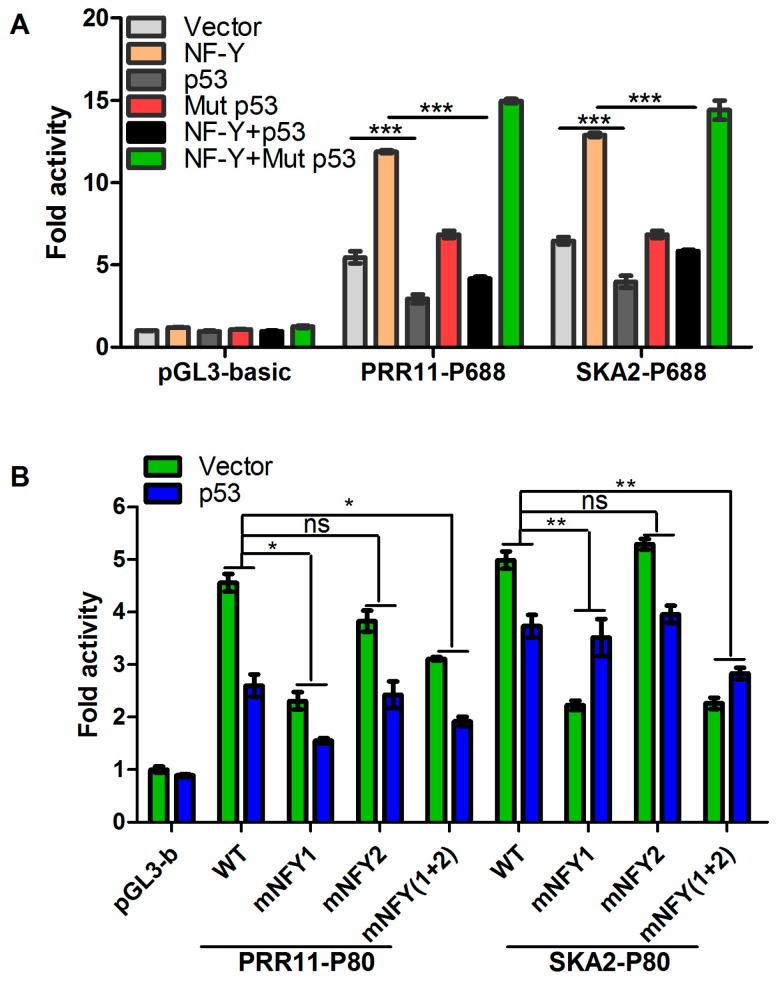
p53 represses the transcriptional activation of the *PRR11-SKA2* bidirectional promoter through NF-Y binding sites. (**A**) p53 overexpression attenuates NF-Y-mediated transactivation of the *PRR11-SKA2* bidirectional promoter. H1299 cells were transiently co-transfected with the indicated luciferase reporter constructs along with *NF-YB*, *p53*, and/or mutant *p53* expression plasmids. Forty-eight hours after transfection, cells were lysed, and luciferase activities were examined as described in Figure 1; (**B**) Disruption of the NF-Y binding sites decreases the repression of the *PRR11-SKA2* bidirectional promoter by p53. Site-directed mutations were introduced into the parental PRR11- or SKA2-P80 (−480/−401) luciferase reporter constructs to disrupt one (mNFY1 and mNFY2) or both (mNFY(1 + 2)) NF-Y binding sites in the *PRR11-SKA2* bidirectional core promoter region. H1299 cells were transiently co-transfected with the indicated luciferase reporter constructs containing wild type or mutated NF-Y binding sites, together with empty or *NF-YB* expression vectors. Forty-eight hours after transfection, luciferase activities were determined as described for Figure 1. * *p* < 0.01, ** *p* < 0.01, *** *p* < 0.001. ns, non-significant.

To determine whether p53 could directly regulate the *PRR11-SKA2* bidirectional transcription unit, the bidirectional promoter region of the *PRR11-SKA2* gene pair was extracted to subject p53 to binding site analysis by using MatInspector professional software. However, the in silico analysis of the *PRR11-SKA2* promoter sequence revealed no consensus p53 binding sites. Notably, we previously demonstrated that *PRR11-SKA2* is a direct target of NF-Y [5]. We thus hypothesized that p53 might repress the *PRR11-SKA2* transcription unit indirectly though interfering with NF-Y. To test this hypothesis, *p53*-defient H1299 cells were transiently co-transfected with PRR11-P688 or SKA2-P688 luciferase reporter constructs along with wild type or mutant *p53* and/or the NF-Y expression vector. The mutant *p53* used here is p53R175H, which lacks transactivation activity but has been shown to have prometastatic gain-of-function in some cancer models. As shown in Figure 3A, the luciferase reporter assay revealed that overexpression of wild type *p53*, but not mutant *p53*, could significantly suppress the basal activity and NF-Y mediated transactivation of the *PRR11-SKA2* bidirectional promoter. Intriguingly, overexpression of mutant *p53* even caused a modest increase in the basal activity and NF-Y-mediated transactivation of the *PRR11-SKA2* bidirectional promoter.

To further determine whether the p53-mediated repression of the *PRR11-SKA2* bidirectional promoter is dependent on the NF-Y binding sites, the wild type PRR11-P80 and SKA2-P80 reporter constructs as well as those with mutation of either or both NF-Y binding sites in the core promoter fragment were co-transfected with the wild type *p53* expression plasmids into H1299 cells. As shown in Figure 3B, the luciferase reporter assay revealed that mutation of the first NF-Y binding site but not the second led to the abolishment of p53-mediated repression of *PRR11-SKA2* promoter activity. Taken together, these results suggest that the first NF-Y binding site is required for the p53-mediated repression of the *PRR11-SKA2* bidirectional promoter.

### 2.4. p53 Reduces the Recruitment of NF-Y to the PRR11-SKA2 Bidirectional Promoter

**Figure 4:**
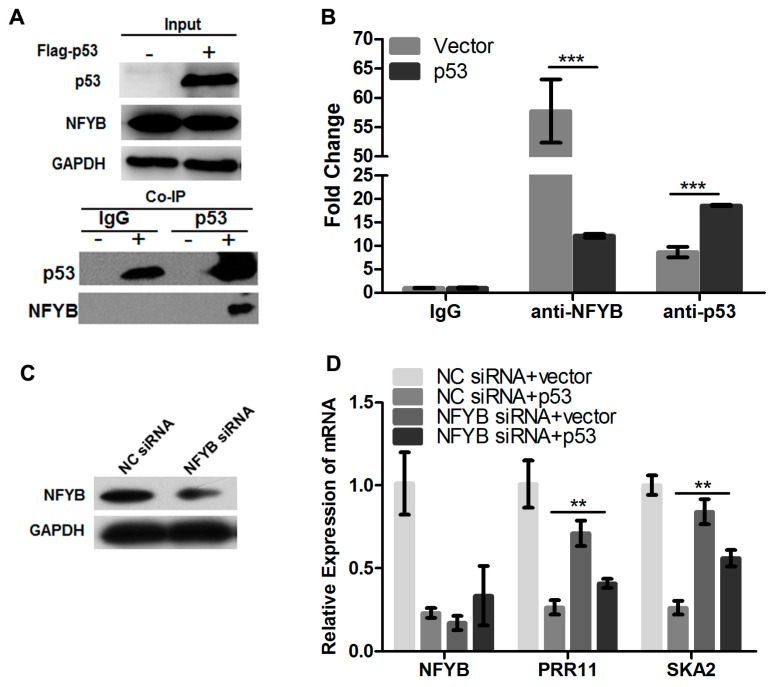
p53 represses *PRR11-SKA2* transcription by decreasing the recruitment of NF-Y on the *PRR11-SAK2* promoter. (**A**) p53 interacts with NF-Y. H1299 cells were transiently transfected with the empty or wild type p53 expression vector. Forty-eight hours after transfection, cells lysates were prepared and immunoprecipitated with anti-p53 antibody followed by Western blotting with antibodies against p53 and NF-YB. Input of p53 and NF-YB was shown by Western blotting; (**B**) p53 overexpression attenuates the binding of NF-Y to the *PRR11-SKA2* bidirectional promoter region. H1299 cells were transiently transfected with the *p53* expression vector. Seventy-two hours after transfection, sheared chromatin was prepared and immunoprecipitated with the indicated antibodies (control IgG, anti-NF-YB or anti-p53). The bound DNA was then isolated and finally subjected to quantitative PCR analysis; (**C**) siRNA-mediated *NF-YB* depletion in H1299 cells; (**D**) *NFYB* knockdown attenuates the repression of *PRR11-SKA2* transcription by p53. H1299 cells were transiently co-transfected with the empty or *p53* expression vectors and/or negative control or *NF-YB*-specific siRNAs. Seventy-two hours after transfection, total RNA was prepared and subjected to qRT-PCR. ** *p* < 0.01, *** *p* < 0.001.

Our previous data showed that NF-Y directly binds to and transactivates the *PRR11-SKA2* bidirectional promoter [5]. We thus deduced that p53 might repress *PRR11-SKA2* transcription by interfering with the binding and transactivation activity of NF-Y on the *PRR11-SKA2* bidirectional promoter. At first, we assessed the association between p53 and NF-Y using a co-immunoprecipitation assay. The results showed that p53 is indeed associated with NF-Y, suggesting that complexes are formed between them in cells (Figure 4A). Furthermore, we conducted a ChIP assay to evaluate the recruitment of p53 and NF-Y to the *PRR11-SKA2* bidirectional promoter region. The results revealed that at the basal condition, NF-Y showed a strong binding activity on the *PRR11-SKA2* bidirectional promoter (Figure 4B). However, when p53 was overexpressed and recruited to the *PRR11-SKA2* promoter region, NF-Y binding was significantly decreased. Thus, these results suggest that p53 may repress *PRR11-SKA2* transcription by reducing the binding amount of NF-Y in the vicinity of the NF-Y binding site in the *PRR11-SKA2* bidirectional promoter.

To further verify the requirement of NF-Y in p53-mediated repression of *PRR11-SKA2*, H1299 cells were transfected with specific siRNA against *NF-YB*, one of the three subunits of NF-Y factor, and/or wild type *p53* expression plasmid. The siRNA-mediated *NF-YB* silencing was confirmed by Western blotting (Figure 4C). The results revealed that down-regulation of the *PRR11* and *SKA2* mRNA levels by p53 overexpression was significantly attenuated with knockdown of *NFYB* as compared with control siRNA transfected cells (Figure 4D). These results suggest that NF-Y is required for the p53-mediated transcriptional repression of *PRR11-SKA2*.

### 2.5. The Clinical Significance of p53-Mediated PRR11-SKA2 Repression in Lung Cancer

**Figure 5:**
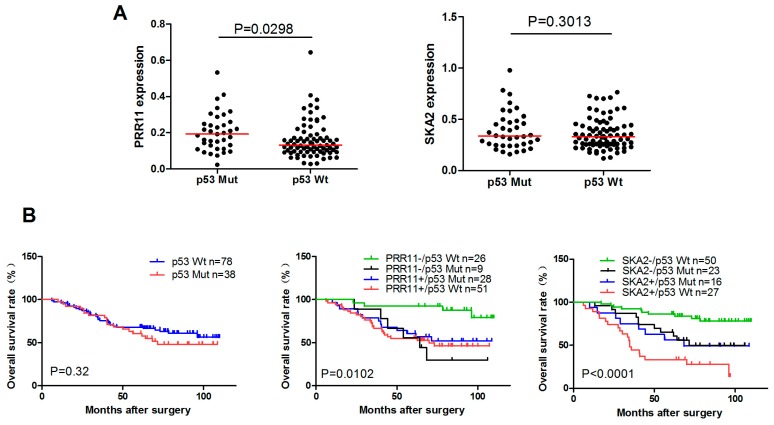
Clinical significance of p53-mediated *PRR11-SKA2* repression in lung cancer. (**A**) Expression levels of *PRR11* and *SKA2* with different *p53* status in lung cancer. The expression levels of *PRR11* and *SKA2* were analyzed in patients with wild type *p53* or mutant *p53* in the Nagoya lung cancer cohort; (**B**) Survival analysis of *PRR11* and *SKA2* expression with different *p53* status in lung cancer. Kaplan–Meier plot of overall survival of lung cancer patients in the Nagoya University cohorts.

Finally, we asked whether p53-mediated *PRR11-SKA2* repression exists and represents clinical significance in lung cancer. For this purpose, we examined the expression levels and prognostic value of PRR11 and SKA2 with different *p53* status in the Nagoya lung cancer cohort in which the p53 status of each patient is available. As shown in Figure 5A, *PRR11* expression is significantly upregulated in the lung cancer-harboring mutant *p53* compared with that harboring wild type *p53*. Intriguingly, probably due to the limited number of samples and functional complexity of *p53* mutations, *SKA2* only showed a slight and statistically non-significant upregulation in lung cancer with mutant *p53* compared with that harboring wild type *p53*. Univariate survival analysis revealed that lung cancer patients with lower expression of either *PRR11* or *SKA*2 along with wild type *p53* exhibited the best overall survival compared with others with *p53* mutation and/or higher expression of either *PRR11* or *SKA2*, whereas only *p53* status was not associated with overall survival (Figure 5B). As p53 represses the *PRR11-SKA2* transcription unit in lung cancer, it is conceivable that patients with lower expression of either *PRR11* or *SKA2* along with wild type *p53* reflect the bona fide functional wild type *p53* and hence show the best outcome.

## 3. Discussion

As a critical tumor suppressor, p53 can be activated by a variety of extracellular and intracellular stresses such as DNA damage and oncogenic signal, and subsequently induces cell cycle arrest, cell senescence and apoptosis [20,21,22]. Notably, as a canonical transcription factor, p53 exerts its function mainly by regulating the expression of various downstream target genes. It is well known that p53 can form tetramers after being activated and then bind to the p53 response element sequence in the promoter region of target genes, thereby directly activating or inhibiting the transcription of target genes [23]. However, reports also demonstrated that some p53 target genes such as *CDC25B*, *cyclin B* and *cdk1* lack consensus p53 response elements. In these circumstances, p53 can indirectly regulate the transcription of target genes by interfering with transcription factors such as NF-Y and Sp1 [24,25].

Our previous study showed that the cancer-related gene pair *PRR11-SKA2* is a novel direct target gene of NF-Y [5]. In the present study, we have for the first time demonstrated that the *PRR11-SKA2* bidirectional transcription unit is negatively regulated by p53 through NF-Y in lung cancer cells, thus placing *PRR11* and *SKA2* on the list of indirect target genes of p53. Based on these findings, we propose the hypothetic working model for this regulatory mechanism. Under the basal condition, NF-Y binds to the CCAAT boxes in the bidirectional promoter region of *PRR11-SKA2* to transactivate the constitutive expression of *PRR11-SKA2*. The tumor suppressor p53 can interact with NF-Y and reduce the binding of NF-Y, preventing the full activation of the *PRR11-SKA2* bidirectional promoter. Thus, wild type *p53* with low expression of *PRR11* and/or *SKA2* lung cancer patients represent a functional regulatory axis of p53-PRR11-SKA2, and hence predicts better prognosis than other groups (Figure 5B). However, when loss of p53 (by inactivation or mutation), one of the most common events in cancer, occurs, inactivated or mutated *p53* is unable to reduce the recruitment of NF-Y to the *PRR11-SKA2* promoter region, eventually leading to the significant upregulation of *PRR11-SKA2* and tumor progression in lung cancer with worse prognosis (Figure 5B). Of note, activation of oncogenes could induce stalling and collapse of DNA replication forks and subsequently lead to formation of DNA double-strand breaks (DSBs), which further causes activation of p53 and hence raises a barrier to tumor progression [26]. Thus, we speculate that, in tumors with wt-*p53*, a possible feedback mechanism involving the activation of the DNA damage response pathway that restrains the oncogenic effect of *PRR11-SKA2* may possibly contribute to the better patient survival found in this study (Figure 5B).

Notably, NF-Y has been shown to interact with coactivators such as histone acetyltransferase p300. Dalvai et al reported that the reduced binding of NF-Y on the *CDC25B* promoter caused by p53 interferes with the recruitment of coactivator p300 [25]. The study also observed that p53 could increase the binding of Sp1 and DNA methyltransferase DNMT1 on the *CDC25B* promoter, suggesting that downregulation of *CDC25B* by p53 is achieved by a switch between the recruitment of coactivator p300 and corepressor DNMT1. Our previous data indicated that the *PRR11-SKA2* bidirectional promoter also contains Sp1 binding sites [5]. Thus, it merits further investigation whether a similar molecular mechanism might be also employed in p53-mediated *PRR11-SKA2* repression. Moreover, Agostino et al. showed that NF-Y interacts with mutant p53 of gain of function, and the resulting mutant p53/NF-Y complexes could bind to NF-Y target genes (such as *cyclin A*, *cyclin B1*, *cdk1* and *cdc25C*) and recruit p300 in response to DNA damage, resulting in aberrant transactivation of the NF-Y target genes and cell cycle deregulation [27]. However, whether mutant p53 binds to NF-Y in a different manner compared to wild-type p53 remains reclusive and needs further investigation. Currently, the potential regulatory role of mutant p53 with respect to the *PRR11-SKA2* transcription unit is under investigation in our laboratory.

## 4. Materials and Methods

### 4.1. Cell Lines

The human lung cancer cell lines, H1299 with deficient *p53* and A549 carrying wild type *p53*, were maintained in a humidified atmosphere containing 5% CO_2_ at 37 °C in RPMI 1640 medium (H1299) or DMEM (A549) supplemented with 100 units/mL penicillin, 100 mg/mL streptomycin, and 10% (*v*/*v*) FBS (Invitrogen, Carlsbad, CA, USA).

### 4.2. Luciferase Reporter Constructs and Reporter Assays

The luciferase reporter constructs including PRR11-P1496, SKA2-P2056, PRR11-P688, SKA2-P688, PRR11-P80 and SKA2-P80 as well as those with mutated NF-Y binding sites were generated as described previously [5]. For luciferase reporter assays, cells were seeded in triplicate into 12-well plates and co-transfected with the indicated reporter plasmids, pRL-TK vector (Promega, Madison, WI, USA) encoding Renilla luciferase and the empty vector or pcDNA3-Flag-p53 expression vector using Lipofectamine^®^ 2000 reagent (Invitrogen). Forty-eight hours after transfection, cells were lysed, and luciferase activity was determined using the Dual-Luciferase assay system (Promega) as described previously [5].

### 4.3. siRNA and Plasmid Transfection

For siRNA transient transfection, siRNAs were transfected into the indicated cells using Lipofectamine RNAiMAX reagent (Invitrogen) according to the manufacturer’s instructions. Cells were then collected and subjected to analysis 24 to 72 h after transfection. The specific siRNAs targeting *p53* and *NFYB*, and negative control siRNAs were chemically synthesized by Shanghai GenePharma (Shanghai, China). The siRNA sequences can be found in Table S1.

For plasmid transient transfection, cells were seeded at a density of 3.5 × 10^5^ cells/6-well tissue culture plate and incubated overnight, followed by transient transfection with the pcDNA3-Flag-p53 expression vector as well as empty control vector using Lipofectamine^®^ 2000 reagent (Invitrogen) according to the manufacture protocols. Cells were then collected and subjected to subsequent analysis 24 to 72 h after transfection.

### 4.4. RNA Isolation and RT-PCR

Total RNA isolation and quantitative qRT-PCR were conducted as described previously [1]. The sequences of the primers used can be found in Table S1.

### 4.5. Western Blotting

The Western blot was conducted as described previously [1]. Briefly, cells were collected and lysed with RIPA buffer supplemented with protease inhibitor Cocktail (Biotool, Houston, TX, USA). The total proteins were quantified using the bicinchoninic acid (BCA) protein assay kit (Thermo Scientific, Beijing, China) and then subjected to SDS-PAGE and immunoblotting. The blots were finally visualized by enhanced chemiluminescence (ECL; Bio-Rad Laboratories, Hercules, CA, USA). The information of the antibodies used in the present study is provided in Table S2.

### 4.6. Co-Immunoprecipitation (Co-IP)

For co-immunoprecipitation experiments, the Flag-p53 expression plasmids was transiently transfected into H1299 cells using Lipofectamine^®^ 2000 reagent (Invitrogen). Forty-eight hours after transfection, the whole cell extracts were prepared and immunoprecipitated with anti-p53 antibody as well as with control IgG. The immunoprecipitated protein complexes were washed three times with IP buffer (Beyotime, Haimen, China) and then analyzed by Western blotting with the indicated antibodies.

### 4.7. Chromatin Immunoprecipitation (ChIP)

ChIP was performed with the EZ ChIP Chromatin Immunoprecipitation kit (Upstate, Lake Placid, NY, USA) as described previously [5]. The sequences of the primers and the antibodies used are provided in Supplementary Tables S1 and S2, respectively. ChIP qRT-PCR data were calculated and expressed as % of recovered immunoprecipitated DNA relative to the Input DNA, i.e., % Input = 100 × 2^−∆*C*t^, where ∆*C*_t_ = (*C*_t[ChIP]_ − (*C*_t[Input]_ − Log_2_100)).

### 4.8. Prognostic Analysis of PRR11 and SKA2 in Lung Cancer Patients

The prognostic value of PRR11 and SKA2 expression was examined in silico using published lung cancer microarray data (GEO database: GSE13213) from the Nagoya University cohort containing 117 stage I–III adenocarcinomas [28]. The microarray data were processed as described previously [29], and Kaplan–Meier analyses were conducted as described previously [1,5]. For Kaplan–Meier analyses, a rational cut-off point was determined by receiver operating characteristic analysis.

### 4.9. Statistical Analyses

All statistical analyses including gene expression analyses in lung cancer samples and overall survival analysis were conducted using the SPSS 16.0 statistical software package (SPSS Inc., Chicago, IL, USA) as described previously [1,5]. *p* < 0.05 was considered statistically significant.

## 5. Conclusions

P53 negatively regulates the expression of the PRR11-SKA2 bidirectional transcription unit through NF-Y in lung cancer cells. Patients with low expression of either PRR11 or SKA2 along with wild type p53 exhibit good prognosis for lung cancer. 

## Supplementary Materials

Supplementary materials can be found at www.mdpi.com/1422-0067/18/3/534/s1.

## Abbreviations

*PRR11*	proline-rich protein 11
*SKA2*	spindle and kinetochore associated complex subunit 2
NF-Y	nuclear transcription factor Y
ChIP	chromatin immunoprecipitation
