# Supplementary material for: The PRR11-SKA2 Bidirectional Transcription Unit Is Negatively Regulated by p53 through NF-Y in Lung Cancer Cells
Source: Int J Mol Sci. 2017 Mar 1;18(3):534. doi: 10.3390/ijms18030534 (PMC5372550; doi:10.3390/ijms18030534)
Supplement: Supplementary file 1 [file ijms-18-00534-s001.pdf]

Supplementary Table S1. Oligonucleotides used in this study

Primers used for RT-PCR analysis

Gene name	Primer sequence
GAPDH	5'-ACCTGACCTGCCGTCTAGAA-3'<br>5'-TCCACCACCCTGTTGCTGTA-3'
PRR11	5′-GACTCCAAAGCTGTGCTTCC-3′<br>5′-CTGCATGGGTCCATCTTTTT-3′
SKA2	5′-ATCCAGTCATTGAAGCCAAACCCCCT-3′<br>5′-CCCGCCTTGATCGCCGTAATGGGTGG-3′
NF-YB	5′-AGGTGCCATCAAGAGAAACG-3′<br>5′-TGTTGTTGACCGTCTGTGGT-3′
p53	5′-GTTCCGAGAGCTGAATGAGG-3′<br>5′-TCTGAGTCAGGCCCTTCTGT-3′

siRNAs used for gene silencing

Name	Sequence
Negative control-siRNA	Sense: 5'-UUCUCCGAACGUGUCACGUTT-3'<br>Antisense: 5'-ACGUGACACGUUCGGAGAATT-3'
P53-siRNA	Sense: 5'-GACUCCAGUGGUAAUCUACTT-3'<br>Antisense: 5'-GUAGAUUACCACUGGAGUCTT-3'
NF-YB-siRNA309	Sense: 5'-GGACAGCAUGAAUGAUCAUTT-3'<br>Antisense: 5'-AUGAUCAUUCAUGCUGUCCTT-3'

Primers used for ChIP analysis

Primer name	Primer sequence
PRR11-ChIP-F1512	5'-GCTGTCACTCAACATCTCGC-3'
PRR11-ChIP-R1631	5'-CGTTTTGTGCCTTTGCCCAA-3'

Supplementary Table S2. Antibodies used in the present study

Protein name	***Maneufacture (cat. number)***	***Applications (working dilution)***	***Website Link***
GAPDH	Xianzhi Bio(AB-P-R 001)	IB(1:5000)	http://www.goodhere.com/showproduct.asp?id=320&classid=34&nid=2
NFYB	Santa Cruz (SC-13045X)	ChIP(1:500)<br>IB(1:2000)	https://www.scbt.com/scbt/product/nf-yb-antibody-fl-207?requestFrom=search
p53	Santa Cruz (SC-126X)	ChIP(1:500)<br>IB(1:2000)	http://www.scbt.com/datasheet-126-p53-do-1-antibody.html
Normal mouse IgG	Milpore (12-371)	ChIP(1:500)	http://www.merckmillipore.com/CN/zh/product/Normal-Mouse-IgG,MM_NF-12-371
Normal rabbit IgG	Milpore (12-370)	ChIP(1:500)	http://www.merckmillipore.com/CN/zh/product/Normal-Rabbit-IgG,MM_NF-12-370
